# Advance Care Planning Website for People With Dementia and Their Family Caregivers: Protocol for a Development and Usability Study

**DOI:** 10.2196/46935

**Published:** 2023-07-26

**Authors:** Fanny Monnet, Charlèss Dupont, Tinne Smets, Aline De Vleminck, Chantal Van Audenhove, Lieve Van den Block, Lara Pivodic

**Affiliations:** 1 End-of-Life Care Research Group Vrije Universiteit Brussel and Ghent University Brussels Belgium; 2 Department of Family Medicine and Chronic Care Vrije Universiteit Brussel Brussels Belgium; 3 LUCAS Center for Care Research and Consultancy KU Leuven Leuven Belgium

**Keywords:** advance care planning, people with dementia, technology, development, usability testing, dementia, caregiver, web-based tools, digital health, user-centered approach

## Abstract

**Background:**

Web-based tools for people with dementia and their family caregivers have considerably increased over the years and offer promising solutions to several unmet needs such as supporting self-care in daily life, facilitating treatment delivery, or ensuring their ability to communicate. The use of web-based tools in the field of advance care planning (ACP) for people with dementia and their family caregivers has yet to be explored and requires careful consideration, given the sensitive topic and the specific needs of people with dementia and their families.

**Objective:**

This paper reports the protocol for a study aiming to develop and simultaneously test the usability of an ACP website designed for, and with, people with dementia and their families.

**Methods:**

The development of the website is based on a process map for the development of web-based decision support interventions and on the Medical Research Council framework for complex intervention development and evaluation. Additionally, we apply a user-centered approach in combination with patient and public involvement (PPI) throughout the development process. We describe our iterative development approach to the website. Participants and a PPI group give feedback on 4 prototypes of the ACP website. For each iteration, we aim to include 12 participants (3 people with dementia, 3 family caregivers, and 3 dyads) in usability testing. In the first 3 iterations, usability testing includes (1) a think-aloud exercise, (2) researcher observations, and (3) the System Usability Scale questionnaire. The last iteration of usability testing is composed of a semistructured interview assessing the layout, content, face validity, and readability of the website. Qualitative data from the think-aloud exercises and interviews are analyzed using thematic analysis. Mean scores are calculated for the System Usability Scale questionnaire.

**Results:**

This study received approval from the Ethical Review Board of Brussels University Hospital of the Vrije Universiteit Brussel. Recruitment began in October 2021. The target date for paper submission of the results of the development and usability testing will be in 2023.

**Conclusions:**

The methods in this protocol describe a feasible and inclusive approach to the development of an ACP website together with people with dementia, their family caregivers, and other stakeholders. We provide a clear overview of how to combine PPI input and user-centered development methods, leading to a transparent and reliable development process. This protocol might stimulate the active participation of people with dementia, their caregivers, and regional stakeholders in future studies on web-based technologies. The results of this study will be used to refine the design and create a relevant and user-friendly ACP website that is ready to be tested in a larger evaluation study.

**International Registered Report Identifier (IRRID):**

DERR1-10.2196/46935

## Introduction

### Background

Web-based tools for people with dementia have considerably increased over the years and offer promising solutions to several unmet needs [1,2]. Many web-based tools have targeted the different needs of people with dementia by, among others, supporting self-care in daily life, facilitating treatment delivery, or facilitating communication [3]. Further, many people with dementia are enthusiastic and positive about using technologies to facilitate their independence [4]. However, the use of web-based tools in the field of advance care planning (ACP) for people with dementia has yet to be explored. To the best of our knowledge, no web-based ACP tool has been developed for people with dementia.

ACP has been defined as an ongoing process that enables individuals to explore and identify their values, reflect upon the meanings and consequences of serious illness scenarios, and define goals and preferences for future care and medical treatment [5,6]. ACP encourages people to discuss these preferences with family and health care providers, appoint a proxy decision maker, and record these preferences and choices [5,6]. We adopt a public health approach to ACP. This approach originated from an ongoing shift in the ACP concept; going from a clinician-led and documentation-focused process that highlights the need of advance directives to a broader concept of ongoing communication among patients, family, and health care providers about several aspects of future care and treatment planning [7,8]. The public health approach to ACP highlights the need to normalize and reconfigure the way decisions are made by reframing ACP as a health-promoting activity through public education and engagement. Underlying this approach is the need to have conversations about end-of-life preferences, death, and dying not only within a medical context (between patients and health care providers) but also within the family context.

Considering the progressive decline in cognitive and functional abilities associated with dementia, ACP can be particularly relevant for people living with dementia, as they become more vulnerable and more dependent on others throughout the disease trajectory [9,10]. Yet, research has shown that ACP is not a widespread practice among people with dementia [11]. People living with dementia and their families are often not well informed about ACP, as they might not be aware of ACP at all or there might be many uncertainties concerning this complex topic, which can be due to a lack of familiarity with the process or with the specific content of ACP [12-14]. Talking about ACP has been found to be complicated as people with dementia and their families experience tensions and perceive this as emotionally difficult conversations [15]. Moreover, people with dementia have quite specific needs in terms of ACP compared to other illnesses. They have a need for a clear understanding of the dementia disease trajectory and what ACP can achieve or not [14]. Furthermore, the more the disease advances and cognition declines (in a nonlinear gradual way), the more family plays a crucial role in ACP in this population [16]. As their condition evolves, many people with dementia may require a surrogate decision maker or legal representative [17].

In other patient groups than dementia, many ACP tools have been developed that are not web-based, such as trainings for professionals or documentation booklets. These have generally aimed to facilitate engagement in ACP discussions by helping patients and family caregivers in reflecting about or making decisions for future care and treatment in coordination with health care professionals [18]. However, web-based tools can have many advantages over paper-and-pencil or face-to-face tools. Web-based ACP tools can be accessed on the internet at any preferred time and place, can be used at everyone’s own pace, with or without the presence of a health care professional or family caregiver, and can reach a larger audience [19]. Additionally, one of the most important features of web-based tools is the use of interactive elements allowing for tailoring to the specific needs and preferences of individuals, which seems particularly relevant for dementia [20]. A recent systematic review identified 10 existing web-based ACP tools published in international peer-reviewed literature. However, they were mostly developed in and limited to the United States [21]. None were designed for people with dementia or tested with people with dementia, despite the specific needs of this population.

### Objectives

In this paper, we describe the protocol of a study that aims to develop and test the usability of an ACP website especially designed for, and with, people with dementia and their families. Furthermore, the website should be user-friendly, interactive, and accessible at any preferred time, so that people with dementia and family caregivers can use the ACP website at their own pace and within the family context.

## Methods

### Development Overview

The methods selected for the development of the ACP website are based on a process map for the development of web-based decision support interventions for a specific audience as proposed by Elwyn and colleagues [22] and follow the Medical Research Council (MRC) framework for the development of complex interventions [23]. Given the complexity of the intervention, combining these approaches is warranted for the development of a website that is both evidence- and theory-informed.

This study focuses on the development stage of the MRC framework for the development of complex interventions [23]. Within the development stage of the MRC framework, we use the process map for the development of web-based decision support interventions, which provides a clear development approach for digital interventions especially. It is composed of main steps such as (1) content specification, with an emphasis on considering patients’ perspectives in addition to synthetizing the current scientific evidence, and (2) creative design, including storyboarding and field and usability testing [22]. Furthermore, as recommended in the MRC framework, we build in theory and integrate continuous stakeholder engagement. Thus, in the first content specification phase, we specify the information that should be included in the ACP website by identifying relevant existing evidence and conducting an assessment of needs among people with dementia, family caregivers, and dementia experts. In the second creative design phase, prototypes of the ACP website are developed and revised in several iterations.

### User-Centered Design and Patient and Public Involvement

In all phases of the development process, we incorporate user views as recommended in recent literature reviews on the development of technological interventions [4,24]. We adopt a user-centered approach throughout the development of the ACP website. User-centered design is a recognized method for complex intervention development [23] and is an evidence-based approach that emphasizes the importance of the needs of end users during the development [25]. Additionally, as advocated by Alzheimer Europe, we integrate meaningful patient and public involvement (PPI) consultation sessions at different stages of the development process to enhance the quality and relevance of the ACP website [26]. PPI sessions are conducted with an advisory group composed of people with dementia, family caregivers, representatives from local dementia associations, and palliative care experts (nurse and consultant for ACP). This group is consulted throughout the development process, in parallel to user testing with study participants, through online meetings and emails.

### Stakeholder Groups Involved in the Development Process

**Figure 1:**
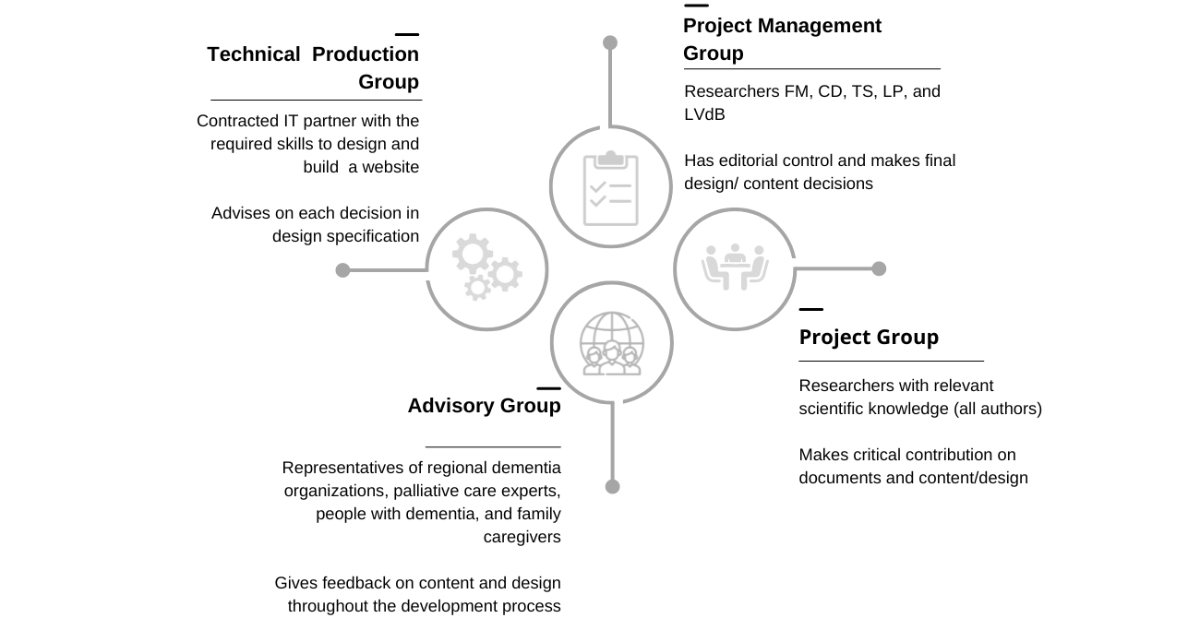
Overview of the stakeholder groups involved in the development process.

The development of the ACP website is supported by 4 groups as suggested by Elwyn et al [22]: a project management group (composed of FM, CD, LVdB, LP, and TS), a project group (composed of all authors of this paper), an advisory group (a PPI group composed of representatives of regional dementia and palliative care organizations and experts in Flanders, people with dementia, and family caregivers), and a technical production group (contracted IT partner). Figure 1 gives an overview of each group’s responsibilities.

### Content Specification Phase of the ACP Website

**Figure 2:**
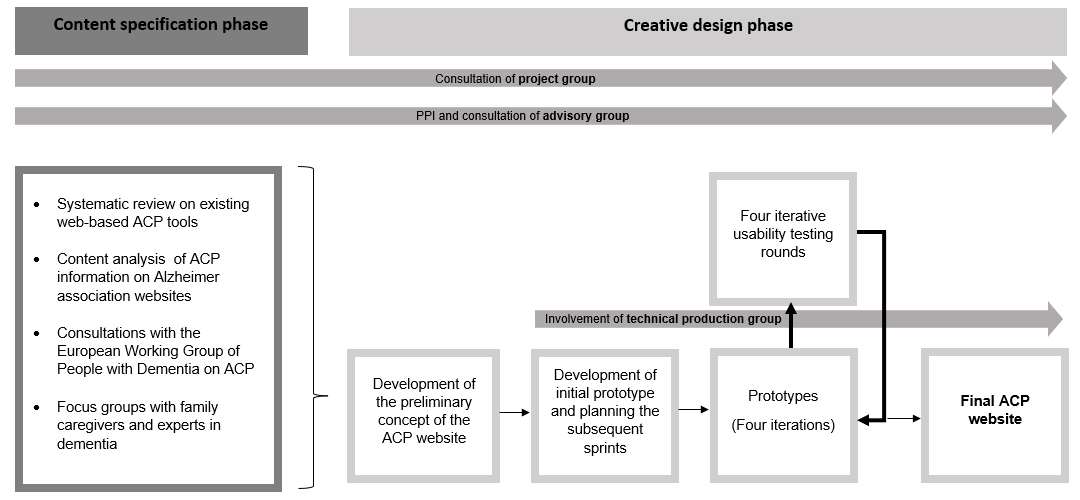
Overview of the development process and usability testing of the ACP website [28,29]. ACP: advance care planning.

In this phase, we specify the information that should be included in the ACP website by identifying relevant existing evidence and conducting an assessment of needs among people with dementia, family caregivers, and dementia experts. An overview of the activities conducted in this phase and their main output is presented in Figure 2. Results from the research activities in the content specification phase are reported separately [27-29]. In this content specification phase, we summarize the evidence from these activities. This includes ensuring the content and structure of the website are in line with previous research on a web-based ACP tool, the information already provided on the internet by dementia associations, and the needs expressed by people with dementia and their families for ACP and an ACP website, as well as with the opinion of experts in the field.

### Creative Design Phase of the ACP Website

Results from the content specification phase inform the design phase of the ACP website. The findings from the evidence base and the user needs assessment are used to build the first concept of the website. An overview of this study, its aims, and all involved groups is shown in Figure 2.

We adopt an agile development approach to build the ACP website. This approach is characterized by an iterative and dynamic development process while collaborating with several groups of stakeholders. It is based on the principles of continuous design improvement and testing based on rapid feedback and change and allows for high-quality adaptive software [30,31]. Such an approach is thus in line with a user-centered approach. Within the agile development approach, we use the Scrum method, as it particularly emphasizes the role of feedback loops. The core characteristic of the Scrum method is the sprint, which is a short development timeframe of approximately 4 weeks [32]. Each sprint requires careful preparation from recruitment of participants to meetings with the advisory group and the technical production group. Based on the complexity of website development and previous work using a Scrum in the development of health-related interventions [33], we estimate that the development of the ACP website will be conducted in 5 successive stages, including 4 sprints. An overview of the sprints is shown in Table 1.

In the presprint, the project management group summarizes and synthetizes the evidence found in the content specification phase. Then, the preliminary concept of the ACP website is discussed in a meeting with the advisory group. The project management group drafts a specification document based on the evidence synthesis and the discussions of the advisory group. Based on this specification document, the technical production group develops the first prototype of the ACP website.

**Table 1:** Overview of the sprints executed during the iterative development process.

Sprints	Objective	Content	User testing	
			Data collection	Participants, n
Presprint	Development of the first prototype	Development of initial prototype Planning of the subsequent sprints	Feedback from advisory group and project group	N/A^a^
Sprint 1	Evaluate the usability of the first 3 features	Usability testing of the home page, structure of the website	Think-aloud method Survey Feedback from advisory group	12 participants (3 people with dementia, 3 family caregivers, and 3 dyads)
Sprint 2	Evaluate usability of the following 3 features	Usability testing of the informational part of the website	Think-aloud method Survey Feedback from advisory group and project group	12 participants (3 people with dementia, 3 family caregivers, and 3 dyads)
Sprint 3	Evaluate usability of the remaining features	Usability testing of the communication part of the website and the web-based tools	Think-aloud method Survey Feedback from advisory group and project group	12 participants (3 people with dementia, 3 family caregivers, and 3 dyads)
Sprint 4	Test the content, face validity, and readability of the tool, and its layout and ease-of-use	Content, face validity, and readability of the whole website	Semistructured interviews Feedback from advisory group and project group	12 participants (3 people with dementia, 3 family caregivers, and 3 dyads)

^a^N/A: not applicable.

### Usability Testing

We follow the definition of the International Organization for Standardization of usability (ISO 9241-11), that is, “how well users can learn and use a product to achieve their goals and how satisfied they are with that process” [34]. This includes the evaluation of the ease of learning, efficiency of use, memorability, error frequency, and satisfaction [35].

People with dementia, family caregivers, and dyads evaluate several versions of the prototype of the ACP website. Based on their input as well as feedback from the advisory group, the technical production group adapts and extends the tool in several iterations (sprints). After 3 usability sprints (sprints 1 to 3), the latest prototype of the ACP website is evaluated by people living with dementia, their main family caregivers, and dyads in terms of content, face validity, readability, and layout (sprint 4). The findings are used to develop the final prototype of the ACP website. An overview of the usability testing is provided in Table 1. The following sections explain the procedure in more detail.

### Participants

We aim to include 48 participants in total. We organize the testing with people with dementia, family caregivers, and dyads. For each iteration (sprint), we aim to include 12 participants (3 people with dementia, 3 family caregivers, and 3 dyads) who are asked to test the prototype and fill in the System Usability Scale (SUS) questionnaire. For every sprint, we seek to enroll new participants. For people with dementia, the inclusion criteria were being aware and informed of their diagnosis, having an interest in and being willing to test an ACP website (including using a computer and the internet), speaking and understanding Dutch, being able to understand the information about this study, and being able to sign a written informed consent form. For family caregivers, the inclusion criteria were as follows: being the main or primary caregiver of a person formally diagnosed with dementia, having an interest in and being willing to test a web-based ACP tool, being 18 years of age or older, and speaking and understanding Dutch.

### Recruitment

We ask participants to use a computer and the internet, which can be challenging, as one needs to have a certain level of computer literacy. The topic of ACP can also be sensitive and can evoke emotional reactions. Therefore, we use a process of active volunteering in our recruitment, that is, potential participants have to indicate themselves if they want to participate in this study. Thus, it is a self-assessment of willingness to participate and interest in the topic.

We recruit participants through different organizations such as the Flemish Alzheimer Liga, memory clinics, or Belgian sickness funds. Individuals who are interested in participating are asked to contact one of the researchers, who in turn sends an information letter and an informed consent form about the study through email. Potential participants are asked to reply to the email or send back the forms if they still want to participate in the study after reading the study information letter and informed consent.

### Data Collection

#### Sprints 1 to 3

The usability tests are conducted in individual sessions with people with dementia, family caregivers, and dyads. Each session is conducted according to a pretested protocol, in the following order: (1) a think-aloud exercise on a set of predefined tasks [36], (2) researcher observations, and (3) a usability questionnaire. The aim of combining these methods is to gather more diverse data. The same type of session is performed with the participants in the sprints 1 to 3. The sessions are conducted in a familiar setting, for instance, the location where they are recruited or in the homes of the participants.

First, we ask the participants to fill in a demographics questionnaire. We give them tasks and oral instructions on how to go through the features of the ACP website prototype. People with dementia, their family caregivers, and the dyads are asked to navigate through each prototype version using a “think-aloud” method. This involves asking participants to verbalize their thoughts, impressions, and feelings while engaging with the tool. A researcher is present to observe and note the participants’ physical cues, successes in tasks, mistakes, difficulties, or comments [36]. Guidance or interference from the researcher is kept to a minimum to investigate whether the ACP website prototype is intuitive. However, if the participants fall silent for too long (eg, if the researcher notices that a participant has silently moved on to a new task or is experiencing difficulties without expressing them), the researcher reminds them to keep thinking aloud using prompts [36].

In addition, participants’ perspectives on usability are further assessed through the SUS questionnaire [37]. The SUS is a widely used and a simple, reliable, and validated 10-item scale that measures subjective usability and that has already been used by people with dementia [36,37]. The SUS score measures users’ perception of the usability of the prototype in terms of effectiveness, efficiency, and satisfaction. Each item is scored on a 5‐point Likert scale (strongly disagree = 1 and strongly agree = 5). Higher scores (range 0‐100) represent better usability [37].

#### Sprint 4

We ask all participants to fill in a demographics questionnaire. Then, we ask people with dementia, family caregivers, and dyads to navigate the ACP website and answer questions in a semistructured interview about the content, face validity, and readability of the website, as well as about its layout.

### Data Analysis

The data from the think-aloud sessions and interviews are audio-recorded and transcribed verbatim. The field notes from the observations serve to further support the data collected during the think-aloud exercise. From sprint 1 to 3, the think-aloud sessions are analyzed through thematic analysis of the notes taken by the researchers and, if the notes were not sufficient, we listen back to the audiotapes to complete the notes. After sprint 4, the data from the transcript are analyzed using thematic analysis [38]. The data are coded by 2 researchers to identify key themes. The researchers meet and discuss the preliminary codes before agreeing on a final list of codes. Disagreements are resolved through discussion.

The data from the SUS questionnaire complements the findings from the think-aloud sessions and interviews. Data are analyzed with SPSS (version 25; IBM Corp). The SUS score is calculated by summing the score contributions of each item. For items 1, 3, 5, 7, and 9, the score contribution is the scale position minus 1. For items 2, 4, 6, 8, and 10, the score contribution is 5 minus the scale position. To obtain the overall value of the SUS score, the sum of the scores must be multiplied by 2.5. Mean scores are calculated, and comparisons between types of participants are considered. A score of 68 or above will be deemed acceptable [39]. Descriptive statistics are used to describe participant demographic characteristics.

### Ethics Approval

This study received approval from the Ethical Review Board of Brussels University Hospital of the Vrije Universiteit Brussel on June 26, 2021 (BUN 1432021000437). To determine the ability of people with dementia to give informed consent, we ask them to read the informed consent form out loud. We ask them per line if they understand their rights and whether they understand what is asked of them. One of the researchers (CD) ensures participants with dementia understand what they sign by discussing the statements formulated in the informed consent form with them. A family caregiver is also present to help the researcher assess the understanding of the person with dementia. If everything is clear, they are asked to sign the informed consent. We also ask the family caregivers to sign the informed consent form of the persons with dementia as witnesses, as recommended by the Alzheimer’s Association National Board of Directors [40]. All data are coded anonymously, and pseudonyms are used when quoting participants.

## Results

We began recruitment of participants in October 2021, and participant enrollment has been completed. A total of 48 participants took part in the usability testing across 4 prototypes of the ACP website, and we conducted a total of 6 advisory group meetings. Data analysis has not started.

Dissemination of the results will be led by the authors and will include presentations at international conferences and publications in scientific peer-reviewed journals and the creation of best practice guidance for the development of technology for people with dementia. Results from the development and usability study will be published in 2023. A larger evaluation study will also be conducted. After evaluation and further adaptation where needed, the ACP website will be made freely available as a resource for people with dementia and their family caregivers, if it is evaluated as acceptable and useful by users, and no negative effects are noticed. The website will be disseminated via the dementia organizations that were involved in our advisory group and is expected to be publicly launched in 2024.

## Discussion

### Principal Findings

User involvement and PPI are recognized as critical components in the development of eHealth and digital solutions, as they can help ensure that interventions meet user needs [41,42]. Involving users in the development of technology has been suggested as an important component for improving technology acceptance, especially for older people [43]. The involvement of people living with dementia in particular has been highly advocated for in the development of technology both in research and by groups such as Alzheimer Europe [24,26,44]. Yet, studies providing a detailed description of user involvement in technology development are scarce. A particular strength of this protocol relates to the step-by-step description of our development process and how we approached the involvement of multiple key stakeholders in all phases of the website development. We particularly emphasize the combination of PPI input and user-centered development methods, leading to a transparent and reliable development process. The development process of the ACP website brings together researchers, regional stakeholders in the fields of dementia and palliative care, as well as people living with dementia and family caregivers to support people with dementia and their families in engaging in ACP.

Furthermore, this protocol describes in detail a unique combination of approaches consisting of the MRC framework for the development of complex interventions [23], the process map for the development of web-based decision support interventions [22], and an agile development approach rooted in user-centered design. The findings of the content specification phase, which are based on rich and extensive input from people with dementia, family caregivers, and dementia experts, as well as up-to-date research evidence on ACP in the field of dementia, have informed the design and content of the ACP website. The iterative development process adopted in the creative design phase facilitated the provision of feedback from the end users. This combination of methods can support researchers and designers in the development of web-based technologies, as well as understanding and considering user needs early on and throughout the development process. We hope that our efforts to describe this research approach will inspire researchers to integrate PPI and user-centered approaches in their own studies. We strongly encourage the active participation of people with dementia, their caregivers, and regional stakeholders in research on the development of web-based technologies for dementia care.

One of the challenges anticipated in the study described in this protocol is that our sample may not be very heterogeneous. In particular, one of the inclusion criteria was to have an interest in testing a website regarding ACP; thus, our sample may be composed of people with generally higher computer skills and who are already familiar with the topic of ACP. Therefore, we may lack different types of perspectives, which could influence the content and design of the ACP website. This study is the first step before a larger evaluation study, which will involve a larger sample and where we will aim for variability in the sample in terms of age, gender, type of dementia, and dyad composition (eg, partners or a parent and child). Additionally, continuous technical support should be organized to encourage the participation of people with lower computer skills.

### Conclusions

The methods put forth in this protocol describe a feasible and inclusive approach to the development of an ACP website. The results from this study will be used to refine the design of the ACP website for a future larger evaluation study to assess the ACP website’s acceptability by people living with dementia and their family caregivers and the effects on people with dementia and family caregivers’ knowledge of ACP, attitudes toward ACP, and intention to engage in ACP. We hope that the findings of the development and usability of the ACP website for people with dementia and their family caregivers will contribute to the design and development of future studies involving the development of web-based technologies for people with dementia.

## Abbreviations

ACP: advance care planning
MRC: Medical Research Council
PPI: patient and public involvement
SUS: System Usability Scale

## Data Availability

Data sharing is not applicable to this paper as no data sets were analyzed for the creation of this study protocol.
